# High Serum Iron level is Associated with Increased Mortality in Patients with Sepsis

**DOI:** 10.1038/s41598-018-29353-2

**Published:** 2018-07-23

**Authors:** Peng Lan, Kong-han Pan, Shuo-jia Wang, Qiu-cheng Shi, Yun-xian Yu, Ying Fu, Yan Chen, Yan Jiang, Xiao-ting Hua, Jian-cang Zhou, Yun-song Yu

**Affiliations:** 1 Department of Critical Care Medicine, Sir Run Run Shaw Hospital, Zhejiang University School of Medicine, Hangzhou, Zhejiang, China; 2 Department of Epidemiology and Health Statistics, School of Public Health, School of Medicine, Zhejiang University, Hangzhou, Zhejiang, China; 3 Department of Infectious Disease, Sir Run Run Shaw Hospital, Zhejiang University School of Medicine, Hangzhou, Zhejiang, China; 4 Department of Clinical Laboratory, Sir Run Run Shaw Hospital, Zhejiang University School of Medicine, Hangzhou, Zhejiang, China

## Abstract

Iron is an essential nutrient for bacterial survival and thus higher iron levels may precipitate bacterial infections. We investigated the association between the serum iron level and prognosis in patients with sepsis by using the single-centre Medical Information Mart for Intensive Care III (MIMIC-III) database. Sepsis patients with iron parameters measured on ICU admission were included and stratified according to quartiles of serum iron levels. A total of 1,891 patients diagnosed with sepsis according to the Sepsis-3 criteria were included in this study, 324 of whom were septic shock. After adjusting for confounding variables, higher iron quartile was associated with an increase in 90-day mortality in the Cox regression analysis. Moreover, a stepwise increase in the risk of 90-day mortality was observed as the quartiles of serum iron levels increased in the patients with sepsis. In conclusion, higher serum iron levels were independently associated with increased 90-day mortality in this large cohort of patients with sepsis.

## Introduction

Iron plays a pivotal role during infection because it functions as a critical cofactor in a variety of physiological metabolic reactions and is an essential nutrient for bacteria. Thus, during bacterial infections, both the host and bacteria compete for iron to thrive. In the human body, most iron is sequestered by haemoglobin^1^. Meanwhile, iron-binding proteins, antibodies and complement proteins in the body function jointly to retain iron^2^. Nevertheless, both free iron and protein-bound iron can be captured by bacteria through various mechanisms, such as siderophores^2,3^, which are secreted by pathogens and possess an affinity to iron that is higher than that of the host transport proteins^4^. Moreover, free iron is toxic because it readily accepts or donates electrons, leading to the generation of reactive oxygen species^5^.

Haemochromatosis, a hereditary iron overload condition, is known to cause susceptibility to infection and iron chelators may reverse this susceptibility^6^. Thus, a higher iron level may precipitate infections. Transferrin saturation (TSAT), a parameter reflects iron availability, was recently reported to be associated with prognosis of ICU patients^7^. Therefore, the likelihood that a higher serum iron level in patients with sepsis is associated with a poorer prognosis is an appealing hypothesis; though, few clinical studies were available as to this hypothesis so far. We hypothesized that higher iron level was associated with worse outcomes in patients with sepsis. Thus, we tested the abovementioned hypothesis by analysing a large database and exploring the association between serum iron levels and the 90-day mortality in patients with sepsis.

## Results

### Characteristics of the study population

A total of 1,891 patients diagnosed with sepsis according to the Sepsis-3 criteria were included in the present study and 324 (17.1%) patients were in septic shock. The baseline characteristics of all patients were presented in Table 1. Half of the patients were male, and approximate two-thirds of the patients were older than 60 years. Most of the patients were admitted from emergency room. One-fifth of the patients were treated by mechanical ventilation upon ICU admission, and the median Simplified Acute Physiology Score (SAPS) II and Sequential Organ Failure Assessment (SOFA) scores were 43 and 5, respectively. With respect to the primary infection sites (Table 2), respiratory, bloodstream and urinary infections accounted for the vast majority of the infections. When it comes to microbiology, *Staphylococcus* (762, 40.3%) and *Escherichia coli* (195, 10.3%) were the two leading pathogens.

**Table 1:** Baseline characteristics and laboratory parameters of the study population.

Characteristics	Total cohort (n = 1891)	Septic shock cohort (n = 324)
Male, n (%)	961 (50.8)	162 (50.0)
Age, n (%)		
16–30	65 (3.4)	4 (1.2)
31–59	581 (30.7)	94 (29.0)
≥60	1245 (65.8)	226 (69.8)
Admission type, n (%)		
Emergency	1809 (95.7)	575 (95.4)
Elective	49 (2.6)	12 (2.0)
Urgent	33 (1.7)	16 (2.7)
Comorbidity, n (%)		
Hypertension	729 (38.6)	114 (35.2)
Malignant neoplasm	422 (22.3)	63 (19.4)
Diabetes Mellitus	591 (31.3)	106 (32.7)
Congestive heart failure	694 (36.7)	123 (38.0)
SAPS II, median (IQR)	43 (35–53)	52 (42–61)
SOFA Score, median (IQR)	5 (3–8)	9 (6–11)
Mechanical ventilation on ICU admission, n (%)	308 (16.3)	64 (19.8)
Adequate antimicrobial therapy, n (%)	1336 (70.7)	240 (74.1)
Tight glucose control, n (%)	1805 (95.5)	304 (93.8)
Hydrocortisone replacement, n (%)	188 (9.9)	76 (23.5)
Laboratory measurements		
Serum creatinine, median (IQR) (mg/dL)	1.3 (0.9–2.2)	1.5 (1.1–2.6)
Lactate, median (IQR) (mmol/L)	1.8 (1.3–2.9)	3.4 (2.5–4.8)
Hemoglobin, median (IQR) (g/dL)	10.7 (9.4–12.1)	11.0 (9.7–12.5)
Transferrin, median (IQR) (mg/dL)	153.0 (119.5–193.5)	144.0 (114.8–186.3)
Ferritin, median (IQR) (ng/mL)	350 (157.0–775.0)	386.5 (191.8–897.8)
White blood cell count, median (IQR) (×10^3^/μL)	11.5 (8.1–16.6)	11.7 (7.4–17.3)

Abbreviations: SOFA score, Sequential Organ Failure Assessment score; SAPS II, Simplified Acute Physiology Score II; IQR, interquartile range.

**Table 2:** Infection origins and pathogens of the study population.

Characteristics	Total cohort (n = 1891)	Septic shock cohort (n = 324)
Site of Infection, n (%)^a^		
Respiratory	1051 (55.6)	188 (58.0)
Bloodstream	831 (43.9)	233 (71.9)
Urinary	741 (39.2)	124 (38.3)
Abdominal	262 (13.9)	51 (15.7)
Gastrointestinal	161 (8.5)	25 (7.7)
Skin and soft tissue	48 (2.5)	6 (1.9)
Pathogenic organism, n (%)		
*Staphylococcus*, *coagulase* (+)	407 (21.5)	71 (21.9)
*Staphylococcus*, *coagulase* (−)	355 (18.8)	62 (19.1)
*Escherichia coli*	195 (10.3)	42 (13.0)
*Pseudomonas aeruginosa*	124 (6.6)	23 (7.1)
*Candida*	120 (6.3)	29 (9.0)
*Klebsiella pneumonia*	121 (6.4)	16 (4.9)
*Streptococcus*	104 (5.5)	19 (5.9)
*Clostridium difficile*	98 (5.2)	13 (4.0)
*Enterococcus faecium*	31 (1.6)	6 (1.9)
*Enterococcus faecalis*	23 (1.2)	7 (2.2)

^a^Patient may have simultaneously multiple sites of infection.

### Outcomes and Cox regression analysis

**Figure 1:**
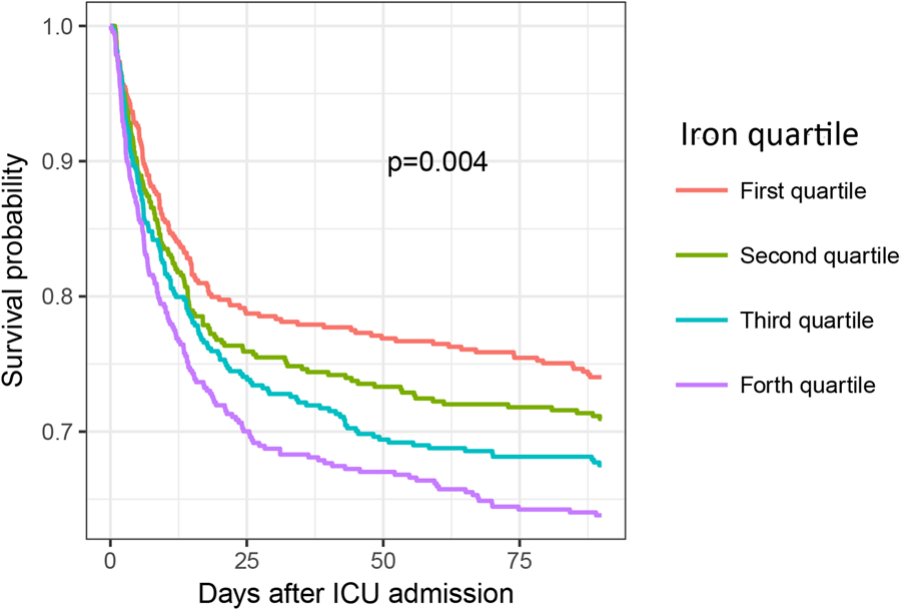
Kaplan-Meier curves showing the association between the iron quartiles and the 90-day mortality.

**Figure 2:**
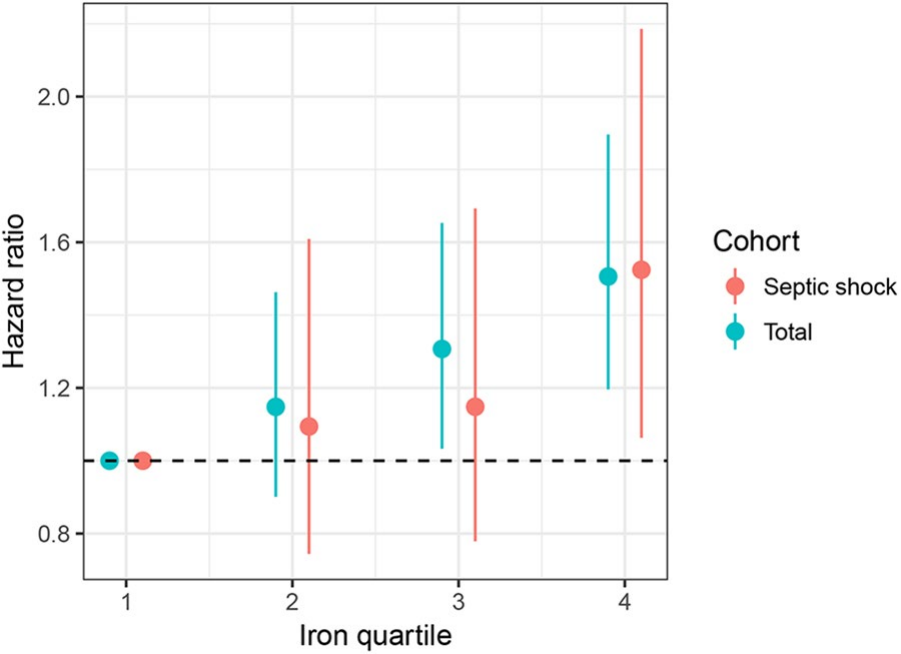
The hazard ratio of iron quartiles with 95% CI for overall population and patients with septic shock. The first iron quartile served as a reference.

The median ICU and hospital stays were 3.8 days and 10.6 days, respectively. The overall 28- and 90-day mortality rates were 21.3% and 30.9%, respectively (Table 3). The mortality rates on days 28 and 90 were significantly increased as the iron quartiles increased (p = 0.001 and p < 0.001 for trend, respectively) (Table 3 and Fig. 1). Cox regression models were established to further determine the independent effect of iron levels on prognosis. After adjusting for possible confounding variables, the risk of death on day 90 increased in a stepwise manner as the iron quartiles increased (Table 4 and Fig. 2). Regarding the other iron parameters, transferrin (HR: 0.997, 95% CI: 0.996–0.999, p < 0.001) and haemoglobin (HR: 0.936, 95% CI: 0.899–0.975, p = 0.001) levels exerted a beneficial effect on the mortality of patients with sepsis, whereas ferritin levels had no significant impact on 90-day mortality (HR: 1.000, 95% CI: 0.999–1.000, p = 0.393) (Table 4). For septic shock cohort, the fourth quartiles of iron was associated with increased risk of death after adjustment for confounding factors (HR: 1.524, 95% CI: 1.063–2.186, p = 0.022, Fig. 2).

**Table 3:** Outcomes of patients according to the quartiles of serum iron.

Outcomes	Total (n = 1891)	First quartile (n = 489)	Second quartile (n = 461)	Third quartile (n = 474)	Fourth quartile (n = 467)	p value
Mortality (n, %)						
90-day	584 (30.9)	127 (26.0)	134 (29.1)	154 (32.5)	169 (36.2)	<0.001
28-day	402 (21.3)	89 (18.2)	89 (19.3)	95 (20.0)	129 (27.6)	0.001
ICU	273 (14.4)	57 (11.7)	62 (13.4)	54 (11.4)	100 (21.4)	<0.001
Hospital	342 (18.1)	73 (14.9)	76 (16.5)	76 (16.0)	117 (25.1)	<0.001
Length of stay (median days, IQR)						
ICU	3.8 (2.0–8.7)	4.3 (2.0–9.0)	3.9 (2.0–8.9)	3.5 (2.0–7.8)	3.6 (2.0–8.7)	0.468
Hospital	10.6 (6.2–18.0)	10.8 (6.5–18.1)	10.6 (6.6–17.8)	10.3 (6.4–17.4)	10.1 (5.8–19.5)	0.670

Abbreviations: ICU, intensive care unit; IQR, interquartile range.

**Table 4:** Cox proportional hazard models exploring the association of iron quartiles with 90-day mortality.

Factors	Univariate model		Multivariate model	
	Hazard ratio (95% CI)	p value	Hazard ratio (95% CI)	p value
Gender (male)	1.217 (1.034–1.433)	0.018	1.067 (0.903–1.260)	0.445
Age				
16–30	Reference	—	Reference	—
31–59	1.762 (0.861–3.606)	0.121	1.383 (0.673–2.842)	0.378
≥60	3.555 (1.767–7.151)	<0.001	2.677 (1.316–5.445)	0.007
Diabetes	0.989 (0.830–1.179)	0.902	—	—
Hypertension	0.828 (0.700–0.982)	0.030	0.872 (0.733–1.039)	0.125
Malignancy	1.879 (1.578–2.238)	<0.001	1.770 (1.480–2.117)	<0.001
Congestive heart failure	1.554 (1.320–1.829)	<0.001	1.433 (1.205–1.704)	<0.001
SOFA score	1.138 (1.114–1.162)	<0.001	1.114 (1.088–1.140)	<0.001
Adequate antimicrobial therapy	0.942 (0.789–1.124)	0.505	—	—
Tight glucose control	0.691 (0.488–0.976)	0.036	0.770 (0.543–1.090)	0.141
Hydrocortisone replacement	1.547 (1.225–1.954)	<0.001	1.009 (0.790–1.287)	0.946
Lactate	1.092 (1.058–1.128)	<0.001	1.076 (1.039–1.115)	<0.001
Hemoglobin	0.926 (0.890–0.963)	<0.001	0.936 (0.899–0.975)	0.001
Creatinine	1.022 (0.990–1.055)	0.182	—	—
WBC	1.002 (0.994–1.011)	0.611	—	—
Transferrin	0.996 (0.995–0.998)	<0.001	0.997 (0.996–0.999)	<0.001
Ferritin	1.000 (1.000–1.000)	<0.001	1.000 (0.999–1.000)	0.393
Iron quartiles				
First	Reference	—	Reference	—
Second	1.148 (0.901–1.463)	0.265	1.149 (0.900–1.468)	0.265
Third	1.307 (1.033–1.653)	0.025	1.309 (1.031–1.660)	0.027
Fourth	1.506 (1.196–1.896)	<0.001	1.506 (1.190–1.908)	<0.001

Abbreviations: WBC, white blood cell; SOFA, Sequential Organ Failure Assessment.

## Discussion

In this large cohort of ICU patients, a higher serum iron quartile was associated with increased 90-day mortality in patients with sepsis. Furthermore, a dose-response analysis indicated that the risk of death on day 90 increased as the quartiles of serum iron levels upon ICU admission increased.

In animal models, bacterial proliferation has been shown to be driven by iron sufficiency and suppressed by iron starvation^8–10^. In a mouse model in which the *hfe* gene was deleted (*hfe*^−/−^), iron abnormally accumulates in tissues. The mortality of the *hfe*^−/−^ mice was approximately doubled after the mice were subjected to caecal ligation and puncture, a typically clinical used animal model of intra-abdominal sepsis^11^. In addition, iron-overloaded mice tended to develop more severe *Salmonella* infections^12^. Undoubtedly, the high iron level was a risk factor in the infected mouse model.

Clinically, during conventional chemotherapy treatment, free iron levels > 2 μM were significantly correlated with a higher risk of sepsis caused by gram-negative bacilli for patients with acute leukaemia^13^. Furthermore, as described in previous studies^6,14^, patients with haemochromatosis, a hereditary disease characterized by an iron overload, were highly susceptible to infections by various pathogens, including *Escherichia coli*, V*ibrio vulnificus*, *Vibrio cholera*, *Klebsiella* species, *Listeria monocytogenes*, and *Shigella* species. Thus, elevated iron level was probably associated with a worse prognosis in patients with sepsis.

The iron level throughout the body is tightly controlled by systems that elaborately regulate iron absorption, systemic transport and cellular uptake and storage^14^. Because nearly all bacteria, fungi and protozoa require a continuous supply of host iron to successfully sustain an infection, humans have evolved powerful strategies, such as the immune system and various iron-binding proteins, to restrict the availability of iron to pathogens^15^. Micro-organisms, such as *Klebsiella pneumonia* and *Escherichia coli*, have developed intricate mechanisms to sequester iron from iron-proteins, such as transferrin, and counter the host’s iron restriction strategies. Siderophores (including aerobactin, enterobactin, salmochelin and yersiniabactin), which are excreted by bacteria, assist the pathogen in acquiring iron^16^. *Neisseria meningitides* can even obtain iron directly from transferrin via receptors, such as TbpA and TbpB, expressed on the bacterial outer membrane^3^. However, the majority of the transferrin that is not saturated with iron possesses a powerful bactericidal or bacteriostatic effect^2^. Transferrin has been recently shown to be a good indicator of organ failure, and higher transferrin level was associated with a decreased 30-day mortality rate in patients with decompensated cirrhosis^17^. Moreover, serum transferrin levels <150 mg/dl were associated with an increased risk of sepsis in burn patients^18^. Consistent with these findings, transferrin was associated with increased survival rates in patients with sepsis in the present study (Table 4).

Although higher iron levels independently facilitate the pathogenesis of infection, some other parameters such as ferritin, the abovementioned transferrin protein and transferrin saturation (TSAT), are associated with iron. In the human body, ferritin, which is regulated by hepcidin, is an iron-binding protein whose function is to store iron in tissue, whereas transferrin in the blood transports iron. Patients who received a kidney transplant and presented ferritin levels ≥500 ng/mL had higher incidence rates (per 1,000 transplant-days) of overall infection (p = 0.017), bacterial infection (p = 0.002), and bloodstream infection (p = 0.011) during the first post-transplant year^19^. Moreover, the one-year infection-free survival rate was significantly lower in these recipients (26% vs 41%; p = 0.004)^19^. As an acute phase reactant, however, ferritin did not show impact on the survival of sepsis (HR: 1.000, 95% CI: 0.999–1.000). The reason might be its complex regulation including growth hormones, hypoxia, anaemia, and endoplasmic reticulum stress response^7^. Moreover, ferritin was defined as the major iron storage protein within hepatocytes and macrophages^20^. The fluctuation of ferritin level was complicated by hepatic disorder, tumour and hematologic instability.

According to a recent prospective study of 155 ICU patients and 156 healthy individuals, TSAT was associated with increased mortality in ICU patients^7^. Actually, TSAT reflects serum iron availability and is highly correlated with iron levels (r = 0.860, p < 0.001) (see Supplementary Table S1). Generally, ICU patients, particularly septic patients, face a katabolic state that decreases iron consumption and increases iron release by destroying erythrocytes and other tissues, and this effect is further amplified by the administration of frequent blood transfusions^7^. Consistent with these findings, the regression and subgroup analyses in our study revealed that iron level was an independent risk factor for 90-day mortality. Moreover, a dose-dependent increase in the risk of death was observed as the iron level increased. The increase in iron-related mortality may be due to excess iron, which directly facilitates recurrent bacterial infections. Moreover, iron catalyses the chemical production of reactive oxygen species, such as the hydroxyl anion and superoxide, and thereby contributes to the development of multiorgan failure^11^. Thus, iron deprivation, i.e., via iron chelators, could be used to manage severe infections. For example, deferoxamine has been assessed as a chemotherapeutic adjuvant treatment in human malaria^21^, and DIBI, another iron chelator, showed a significant curative effect on animal models of sepsis^22^.

Although this study is a large cohort study designed to explore the relationship between the serum iron levels and outcomes in patients with sepsis, our study has several limitations. Firstly, the study was based on a publicly accessible, single-centre database, which may lead to concerns regarding the generalizability of the conclusions and the confounding bias caused by the missing data. However, this database has been explored by many authors worldwide, and articles have been published guaranteeing the data quality^23,24^, potentially facilitating the generalization of our findings. Secondly, we included only patients with sepsis for whom iron measurements were available, which may be responsible for some selection bias. Thirdly, we adopted only one measurement of the iron parameters on ICU admission rather than the trends, which may ignore the effect of iron levels on the trends. Fourthly, we could only extract the administration date rather than exact hours of antimicrobials in the database. Thus, we could not incorporate the time to start of antimicrobials to the Cox regression analysis. Finally, the database was relative old (patients between 2001 and 2012) and some management strategies have been improved for sepsis recently.

In summary, higher serum iron level was independently associated with an increased 90-day mortality in patients with sepsis.

## Methods

### Data source

This study was based on a publicly accessible critical care database named Medical Information Mart for Intensive Care III (MIMIC-III, version 1.4), which is a large, single-centre database containing information of 46,520 patients (aged 16 years or older) who were admitted to Beth Israel Deaconess Medical Center (a teaching hospital of Harvard Medical School in Boston, Massachusetts) between 2001 and 2012^25^. The database contains data pertaining to general information (i.e., demographics, billing, ICD-9 codes, ICU types, SOFA scores, SAPS II scores, etc.), treatment process (i.e., medications, procedures, laboratory tests, fluid balance, image reports, etc.) and survival data. The data were extracted from the MIMIC-III database using structure query language (SQL) with pgAdmin4 PostgreSQL 9.6.

### Study patients and definitions

All patients with sepsis for whom measurement of iron parameters were available after admission to the ICU were included in the analysis. Infectious patients were defined as individuals meeting one of the following criteria: (1) ICD-9 diagnostic code containing the terms “infection”, “pneumonia”, “meningitis”, “peritonitis”, “bacteraemia”, “sepsis” or “septic”, or (2) a positive microbiological culture^26^. According to the Sepsis-3 definition, patients with infections or suspected infections and an increase of SOFA score of 2 points or more were defined as sepsis^27^. Furthermore, patients with sepsis who required vasopressors to maintain a mean arterial pressure of 65 mm Hg or greater and with serum lactate level greater than 2 mmol/L in the absence of hypovolemia were categorized as septic shock^27^. Patients who received iron supplementation during the ICU stay were excluded.

For some patients for whom multiple iron measurements were available, the first measurement after ICU admission was used. Other data, including gender, age (categorized into 16–30, 31–59, and 60 years or above), admission type (elective, emergency and urgent), use of mechanical ventilation upon ICU admission, comorbidities, infection sites, pathogenic organisms, creatinine level, lactate level, white blood cell count, and SAPS II scores were extracted. Adequate antimicrobial treatments and tight glucose control were defined as previously described^28,29^. Missing values were imputed as the mean values, because all variables included less than 10% of missing observations. Patients were stratified according to the quartiles of serum iron level.

The primary outcome was all cause 90-day mortality. The secondary outcomes included 28-day mortality and the length of the hospital and ICU stays.

### Statistical analysis

Shapiro-Wilk tests were performed and histograms were generated to determine the normality of the distribution of the continuous variables. Normally distributed continuous variables were reported as the means ± SD, whereas skewed variables were summarized as the median and interquartile range (IQR). Categorical variables were compared using a chi-square analysis or Fisher’s exact test to determine the differences among the quartiles. For comparisons among the four quartiles of iron level, analysis of variance (ANOVA) or the Kruskal-Wallis test was performed. Outcome data were compared using a trend analysis.

We compared the survival rates using log-rank tests and present the results as Kaplan–Meier curves. We developed multivariable Cox proportional hazard models for the overall population and patients with septic shock to determine the independent effect of the quartiles of serum iron level on 90-day mortality. Variables with p < 0.05 in the univariate analysis were further incorporated into multivariate Cox proportional hazard models. Iron parameters with a moderate to strong correlation strength (r > 0.3) were not simultaneously entered into the multivariable models to avoid collinearity (see Supplementary Table S1). The hazard ratios and their 95% CIs were calculated. All analyses were performed using R 3.3.3, and a p-value less than 0.05 was considered statistically significant.

### Data availability

The data analysed in the study are fully available on the website.

## Electronic supplementary material

Supplementary Information
