# Supplementary material for: High Serum Iron level is Associated with Increased Mortality in Patients with Sepsis
Source: Sci Rep. 2018 Jul 23;8:11072. doi: 10.1038/s41598-018-29353-2 (PMC6056487; doi:10.1038/s41598-018-29353-2)
Supplement: Supplementary file 1 — Supplementary Information [file 41598_2018_29353_MOESM1_ESM.docx]

**High Serum Iron level is Associated with Increased Mortality in Patients with Sepsis**

Peng Lan^1,*^, Kong-han Pan^1,*^, Shuo-jia Wang^2^, Qiu-cheng Shi^3^, Yun-xian Yu^2^, Ying Fu^4^, Yan Chen^3^, Yan Jiang^3^, Xiao-ting Hua^3^, Jian-cang Zhou^1^ and Yun-song Yu^3^

^1^Department of Critical Care Medicine, Sir Run Run Shaw Hospital, Zhejiang University School of Medicine, Hangzhou, Zhejiang, China; ^2^Department of Epidemiology and Health Statistics, School of Public Health, School of Medicine, Zhejiang University, Hangzhou, Zhejiang, China; ^3^Department of Infectious Disease, Sir Run Run Shaw Hospital, Zhejiang University School of Medicine, Hangzhou, Zhejiang, China; ^4^Department of Clinical Laboratory, Sir Run Run Shaw Hospital, Zhejiang University School of Medicine, Hangzhou, Zhejiang, China

^*^ These authors contributed equally to this work.

**Correspondence to**: Dr.Jian-cang Zhou, Department of Critical Care Medicine, Sir Run Run Shaw Hospital, No.3 Qing Chun Road East, Hangzhou, Zhejiang 310016, People’s Republic of China. E-mail: [jiancangzhou@zju.edu.cn](mailto:jiancangzhou@zju.edu.cn).

| **Iron parameters** | **Iron** | **Ferritin** | **TIBC** | **Transferrin** | **TSAT** | **UIBC** |
| --- | --- | --- | --- | --- | --- | --- |
| Iron | - | 0.149  (p<0.001) | 0.088  (p<0.001) | 0.088  (p<0.001) | 0.860  (p<0.001) | -0.389  (p<0.001) |
| Ferritin | 0.149  (p<0.001) | - | -0.319  (p<0.001) | -0.319  (p<0.001) | 0.271  (p<0.001) | -0.365  (p<0.001) |
| TIBC | 0.088  (p<0.001) | -0.319  (p<0.001) | - | 1.000  (p<0.001) | -0.281  (p<0.001) | 0.883  (p<0.001) |
| Transferrin | 0.088  (p<0.001) | -0.319  (p<0.001) | 1.000  (p<0.001) | - | -0.281  (p<0.001) | 0.883  (p<0.001) |
| TSAT | 0.860  (p<0.001) | 0.271  (p<0.001) | -0.281  (p<0.001) | -0.281  (p<0.001) | - | -0.665  (p<0.001) |
| UIBC | -0.389  (p<0.001) | -0.365  (p<0.001) | 0.883  (p<0.001) | 0.883  (p<0.001) | -0.665  (p<0.001) | - |
| Table S1. Correlation coefficients of iron parameters in overall population. Abbreviation: TIBC, Total Iron Binding Capacity; TSAT, transferrin saturation; UIBC, Unsaturated Iron Binding Capacity | | | | | | |
